# Additional diagnostic value of ratio indices of quantitative contrast-enhanced ultrasound parameters in small solid C-TIRADS 4 thyroid nodules

**DOI:** 10.3389/fonc.2025.1565400

**Published:** 2025-04-22

**Authors:** Jiayi Xie, Wengang Liu, Jinguang Zhou, Ping Zhou

**Affiliations:** Department of Ultrasound, Third Xiangya Hospital, Central South University, Changsha, Hunan, China

**Keywords:** contrast-enhanced ultrasound (CEUS), quantitative, qualitative, thyroid nodule, thyroid papillary carcinoma, Chinese thyroid imaging reporting and data system(C-TIRADS)

## Abstract

**Background:**

To investigate the efficacy of contrast-enhanced ultrasound (CEUS) parameters, particularly ratio indices of quantitative CEUS parameters, for differentiation of small solid C-TIRADS 4 thyroid nodules.

**Materials and methods:**

235 small solid C-TIRADS 4 thyroid nodules with determinate pathological results, including 175 nodules in the training cohort and 60 nodules in the validation cohort were retrospectively evaluated. The ratio indices of the internal tissue to peripheral tissue and the internal tissue to healthy tissue of quantitative parameters were calculated. In the training cohort, the meaningful quantitative ratio indices with an AUC > 0.7 and qualitative parameters were further included in multivariate regression analysis. The diagnostic efficacy of the logistic model was evaluated.

**Results:**

In single-factor analysis, C-TIRADS, enhancement degree, mTTI ratio (L/P), TTP ratio (L/P), WiR ratio (L/P), WoR ratio (L/P) and TTP ratio (L/H) were significant parameters for differentiation of thyroid nodules (P < 0.05). The multifactor analysis showed that there was significant difference in C-TIRADS, mTTI ratio (L/P), TTP ratio (L/P), WiR ratio (L/P), WoR ratio (L/P) and TTP ratio (L/H) between two groups (P < 0.05). The logistic model was generated, and the AUC, sensitivity, specificity, and accuracy of the training cohort were 0.935(95% CI: 0.888–0.967),85.71%, 88.57%, and 86.86%, respectively. The logistic model demonstrated significantly higher diagnostic performance compared to individual parameters (P < 0.001). In the validation cohort, the diagnostic model had an AUC of 0.910,sensitivity of 87.9%, specificity of 92.6%, and accuracy of 90.25%.

**Conclusion:**

Ratio indices of quantitative parameters have high diagnostic values in differentiating small solid thyroid nodules. Combining C-TIRADS with both qualitative and quantitative CEUS parameters enhances the diagnostic accuracy of malignant thyroid nodules.

## 1 Introduction

The progression and utilization of imaging technologies and equipment have led to a significant increase in the detection rate of thyroid nodules over the past several decades (1). Since the incidence of malignancy of thyroid nodules is relatively low and many malignant thyroid nodules usually have no obvious clinical manifestations (2), accurate diagnosis of malignant nodules is of great significance for clinicians. Ultrasound (US) is often used as a routine examination of thyroid nodules due to its non-invasive and convenient characteristics (1). The Chinese Thyroid Imaging Reporting and Data System (C-TIRADS) has delineated five principal sonographic characteristics to aid radiologists in differentiating between benign and malignant thyroid nodules (3). However, the diagnosis of nodules classified as C-TIRADS 4 has a malignancy rate ranging from 2% to 90%, which makes the decision of whether to perform invasive operation such as fine needle aspiration (FNA) or even surgery controversial (4). Although papillary thyroid microcarcinoma (PTMC), defined as papillary thyroid carcinoma with a maximum diameter of ≤1 cm, generally exhibits indolent growth and a relatively favorable prognosis, 30%-40% of PTMC may have tumor biological variation due to gene mutation and demonstrate aggressive characteristics, including extrathyroidal extension (ETE), central lymph node metastasis (CLNM), and distant metastasis (5, 6). A previous study demonstrated that PTMC with metastasis needs more aggressive treatment options such as lymphadenectomy and radioactive iodine treatment (7). Moreover, microwave (MW) ablation has been recommended for PTMC, providing a potential minimally invasive treatment option to avoid the occurrence of aggressive manifestations (8). Thus, there is a need for supplementary technologies to enhance accuracy efficiency of thyroid nodules.

Contrast−enhanced ultrasound (CEUS) is an advanced technique that extends from conventional ultrasound, having its advantage of hemodynamically assessing the microvascular distribution of lesions and parenchymal perfusion (9). Recent studies on CEUS have reached a consensus that characteristics such as delayed wash-in time, rapid wash-out rate, hypo-enhancement, heterogeneous enhancement, and centripetal enhancement are significant indicators of malignancy (4, 10, 11). Some previous studies have also found that homogeneous hyper-enhancement peripheral ring was a significant indicator of benignity, and irregular hypo- or hyper-enhancement peripheral was more frequently observed in thyroid nodules (12–14). However, the contribution of qualitative CEUS features is still controversial, especially about the peripheral of thyroid tissue. First, the small size of solid thyroid nodules often exhibits a lack of vascularization and complex microvascular variations (8), which can render the distinctions in enhancement characteristics among the internal tissue of the thyroid nodule, the peripheral tissue of the nodule, and healthy thyroid tissue too subtle for visual identification. In addition, qualitative CEUS examination largely depends on radiologists’ experience and subjectivity (15). Therefore, there is a pressing need for further advancements in CEUS technology.

Recently, quantitative analysis of CEUS has been widely used in the evaluation of variate organs including the liver, pancreas, breast, and kidney (16). The quantitative analysis automatically generates a time-intensity curve (TIC), from which a series of quantitative parameters are derived. These parameters provide a more comprehensive digital representation of the microvascular density of the lesions (8, 9, 17, 18). However, most previous studies have predominantly focused on the quantitative parameters of the internal tissue of thyroid nodules and healthy thyroid tissue on CEUS. These studies often analyzed only the absolute values of each parameter, which may result in inconclusive findings due to the limited scope of quantitative parameters, variability in contrast agents, and discrepancies in the quantitative analysis software employed (9, 19, 20). In standard conventional and contrast-enhanced ultrasound examinations, comparisons between the nodule and the surrounding perinodular tissue have been routinely conducted, and some previous studies have focused on the comparison of CEUS quantitative parameters in internal thyroid nodules with healthy thyroid tissue (8, 21). However, the quantitative CEUS features of the peripheral tissue of thyroid nodules were not deeply investigated.

Therefore, the objective of this study was to investigate the additional diagnostic value of ratio indices of qualitative CEUS parameters among the internal tissue of thyroid nodules, the peripheral tissue of thyroid nodules and healthy thyroid tissue in differentiation of small solid thyroid nodules categorized as C-TIRADS 4 and compare their diagnostic efficacy with that of C-TIRADS and qualitative CEUS features.

## 2 Materials and methods

### 2.1 Study subjects

We reviewed the CEUS database from November 2020 to February 2023 at The Third Xiangya Hospital of Central South University. Ethical approval for this study (IRB of Third Xiangya Hospital, Central South University 2021-S222) was provided by the Ethical Committee IRB of Third Xiangya Hospital, Central South University. The inclusion criteria were as follows: (1) patients aged 18 years or older with solid or predominantly solid thyroid nodules measuring maximum diameter ≤ 1.0 cm, classified as C-TIRADS 4;(2) a video acquisition time of CEUS is no less than 90 seconds; (3) nodules with accurate pathological results of papillary carcinoma,or benign results proved by repeated FNAs; (4) patients who had not undergone needle biopsy or radiotherapy before US and CEUS examinations. The exclusion criteria included: (1) a video acquisition time of CEUS that was too short; (2) nodules mostly occupied by coarse calcification or necrosis; (3) patients who were unable to cooperate during CEUS procedure, rendering the CEUS video unqualified.

In total, 98 benign thyroid nodules from 89 patients and 137 malignant nodules from 130 patients were included.

### 2.2 B-mode US and CEUS qualitative analysis

All ultrasound examinations were conducted by two radiologists, one with 3 years of experience and the other with 20 years of experience in CEUS imaging diagnosis, using one high-resolution ultrasound machine (Acuson Sequoia [Siemens Healthineers, Erlangen, Germany]) equipped with an L9-4 linear-array transducer.

Thyroid nodules were classified according to C-TIRADS classification as follows:

Benign features of nodules include pure cystic or spongy component, and point-like strong echo with “coma tail” artifact (-1 point).

Suspected malignant features of nodules include taller-than-wide shape (+1 point), solid component with hypoechogenic (+1 point), markedly hypoechogenic (+1 point), microcalcification (+1 point), and blurred edges or extra-thyroid invasion (+1 point).

C-TIRADS 1: normal thyroid, rate of malignancy = 0%

C-TIRADS 2: rate of malignancy = 0% (-1 point)

C-TIRADS 3: rate of malignancy <2% (0 point)

C-TIRADS 4a: rate of malignancy 2%-10% (1 point)

C-TIRADS 4b: rate of malignancy 10%-50% (2 points)

C-TIRADS 4c: rate of malignancy 50%-90% (3 or 4 points)

C-TIRADS 5: rate of malignancy > 90% (5 points)

In this study, C-TIRADS ≥4b was set as a cutoff to predict malignancy.

CEUS was performed following the intravenous injection of 2.4ml of sulfur hexafluoride microbubbles (SonoVue^®^, Bracco, Milan, Italy) via the cubital vein, followed by a 5 ml saline flush. All CEUS examinations were conducted by the radiologist with over 20 years of experience. A video not less than 90s in length was recorded continuously and steadily throughout the procedure to document the enhancement process of the thyroid nodule. In cases where patients presented with more than one suspicious nodule, an additional CEUS examination was performed after 10 minutes with another injection of SonoVue microbubbles. The CEUS data were exported in DICOM format as a cine loop. The radiologist with over 20 years of experience in CEUS imaging diagnosis conducted the qualitative analysis. The qualitative CEUS features included: enhancement degree (hypo-enhancement, hyper- or iso-enhancement), enhancement homogeneity (homogeneous or heterogeneous), and enhancement pattern (centripetal or centrifugal enhancement).

### 2.3 CEUS Quantitative analysis

**Figure 1:**
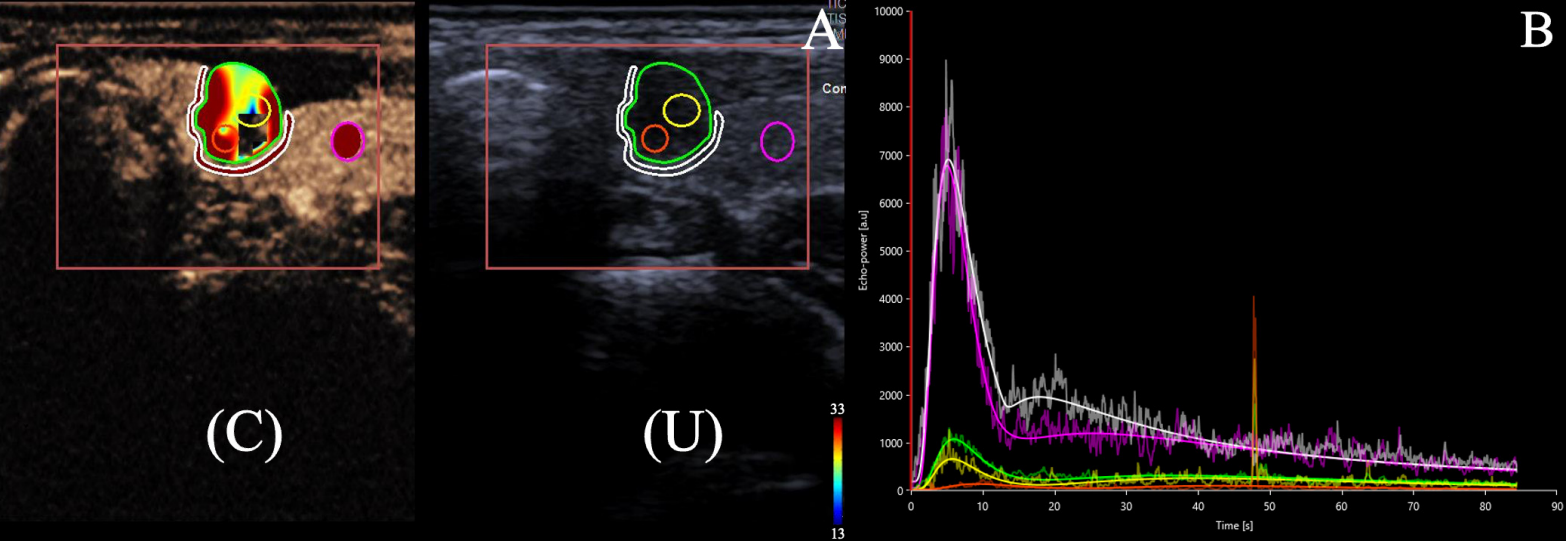
**(A)** 22-year-old female with a 6.7 × 5.9 × 7.0 mm nodule on the left thyroid lobe. The nodule has solid component with hypoechoic and blurred edge, which was categorized as C-TIRADS 4b. Surgical pathology showed a papillary thyroid carcinoma. **(A)** Dual US and CEUS image of the internal tissue of thyroid nodule (ROI 1, green circle outlining the entirety of the nodule, ROI 2 and 3, yellow and orange circles randomly selected to represent the internodular contrast-enhanced areas), the peripheral tissue of thyroid nodule(ROI 4, white circle surrounding the nodule),and the healthy tissue of the gland (ROI 5, pink circle set in the healthy thyroid tissue) under CEUS (C) and conventional ultrasound (U). **(B)**. Time-intensity curve (TIC)s for the internal tissue (green, yellow, and orange lines), the peripheral tissue of the thyroid nodule (white line) and the healthy tissue of the gland (pink line) were generated. The lesion ROI curves were lower than the reference ROI curves, indicating hypoperfusion.

A quantitative analysis was conducted by a trained radiologist using VueBox^®^ software (Bracco, Italy). The major steps involved were: 1) selecting the arrival time and eliminating irrelevant clips; 2) identifying the fragments indicative of peak enhancement and delineating three distinct kinds of region of interest (ROIs). a. Within the nodule tissue, two or three ROIs were established: one ROI was designated to encompass the entirety of the nodule, while the remaining one or two ROIs were randomly selected to represent the contrast-enhanced areas within the nodule. b. The second ROI was defined as a strip along the maximum inner radial direction of the nodule, with a width of approximately 1 to 2 mm. c. The third ROI was delineated within the healthy thyroid tissue, positioned at the same depth as the lesion (**Figure 1**); 3) utilizing the motion compensation function provided by VueBox^®^ to mitigate imaging instability caused by patient respiration during the examination. A time-intensity curve (TIC) and quantitative parameters were generated as follows: 1) peak enhancement (PE), defined as the intensity at peak enhancement; 2) wash-in area under the curve (WiAUC), defined as the area under the curve from the time of arrival to peak enhancement; 3) rise time (RT), defined as the duration for the contrast agents to ascend from 10% to 90% of peak enhancement; 4) time to peak (TTP), defined as the time taken for the mass’s contrast intensity to rise to peak enhancement; 5) wash-in rate (WiR), defined as a tangent at the ascending segment of the curve; 6) wash-in perfusion index (WiPI), defined as the ratio of WiAUC and rise time; 7) mean transition time (MTTI), defined as the time between the point of arrival of the contrast agent and the point of clearance; 8) wash-out area under the curve (WoAUC), defined as the area under the curve from the time of peak enhancement to clearance;9) wash-out rate (WoR), defined as a tangent at the descending segment of the curve. When there was no calcification or necrotic area in the nodule, these quantitative parameters generated by the ROI of whole nodule were included; when there was calcification or necrotic area in the nodule, the average value of parameters of two ROIs set in the contrast-enhanced areas within the nodule were calculated and included.

Subsequently, we calculated the ratios of these quantitative parameters among the internal nodule, peripheral nodule, and healthy thyroid tissue. L/H means the internal tissue of the lesion/the healthy tissue of the thyroid; L/P means the internal tissue of the lesion/the peripheral tissue of the thyroid nodule.

### 2.4 Statistical analysis

The statistical analysis was conducted using SPSS version 26.0. Quantitative variables were expressed as mean ± standard deviation or median (interquartile range) as appropriate. The Pearson Chi-Square test or Fisher’s exact test was used for qualitative data. The Kolmogorov-Smirnov (K-S) test was used for assessing whether the quantitative data followed a normal distribution. Independent sample t-tests and paired sample t-tests were used for quantitative data that adhered to a normal distribution, while the Wilcoxon Mann-Whitney test was used for quantitative data that did not conform to normality. Significant variables identified through univariate analysis were subsequently incorporated into logistic regression analysis. The receiver operating characteristic (ROC) curve was used for establishing the diagnostic threshold. The Z test was used for comparing the AUC of above parameters. A P-value of less than 0.05 was deemed statistically significant.

## 3 Results

### 3.1 Demographics of the thyroid nodules in the training cohort

A total of 169 patients (136 females and 33 males) participated in this study. The ages of the patients varied from 22 to 73 years, and there was a significant difference in age between patients with benign nodules and those with malignant nodules (P= 0.002). The size of the thyroid nodules showed no significant difference between benign (median size: 8.00 mm [IQR: 5.90, 10.00]) and malignant (median size: 7.00 mm [IQR: 5.30, 9.65]) nodules (P = 0.099). The distribution of nodules was as follows: 80 nodules (45.7%) were located in the left lobes, 78 nodules (44.5%) in the right lobes, and 18 nodules (9.8%) in the isthmus, with no significant difference in laterality (P = 0.310).

According to the Chinese Thyroid Imaging Reporting and Data System C-TIRADS) classification,41,85,49 recruited nodules were classified in category 4a,4b, and 4c, respectively. In this study, 24.4% of C-TIRADS 4a nodules were proved to be malignant and 75.6% were benign, and 70.8% of C-TIRADS 4b and 4c nodules were proved to be malignant and 29.2% were benign. There was a significantly difference in C-TIRADS between malignant and benign nodules (P< 0.001).

### 3.2 Single factor analysis results of CEUS qualitative parameters of thyroid nodules in the training cohort

The single factor analysis results of US and CEUS qualitative parameters were summarized in **Table 1**. Hypo-enhancement was more commonly observed in malignant nodules compared to benign nodules (P< 0.001), while heterogeneous enhancement (P = 0.354) and a centripetal enhancement pattern (P= 0.094) did not demonstrate significant differences between the two groups.

**Table 1:** Demographics and US and CEUS qualitative parameters of thyroid nodules in the training cohort.

Parameter		Overall (n=175)	Malignant (n=105)	Benign (n=70)	P value
Age,y	<45	112 (63%)	28 (26.7%)	35 (50%)	0.002
	≥45	63 (37%)	77 (73.3%)	35 (50%)	
Sex	Male	35 (20%)	19 (18.1%)	16 (22.9%)	0.440
	Female	140 (80%)	86 (81.9%)	54 (77.1%)	
Size,mm		7.40 (5.60,10.0)	7.00 (5.30,9.65)	8.00 (5.90,10.00)	0.099
Laterality	Left	80 (45.7%)	48 (45.7%)	32 (45.7%)	0.310
	Right	78 (44.6%)	44 (41.9%)	34 (48.6%)	
	Isthmus	17 (9.7%)	13 (12.4%)	4 (5.7%)	
C-TIRADS	4a	41 (23.4%)	10 (9.5%)	31 (44.3%)	<0.001
	4b and 4c	134 (76.6%)	95 (90.5%)	39 (48.6%)	
Enhancement degree	Hypo-enhancement	117 (66.9%)	84 (80.0%)	33 (47.1%)	<0.001
	Hyper-/Iso- enhancement	58 (33.1%)	21 (20.0%)	37 (52.9%)	
Enhancement homogeneity	Homogeneous	90 (51.4%)	51 (48.6%)	39 (55.7%)	0.354
	Heterogeneous	85 (48.6%)	54 (51.4%)	31 (44.3%)	
Enhancement pattern	Centripetal	166 (94.9%)	102 (97.1%)	64 (91.4%)	0.094
	Centrifugal	9 (5.1%)	3 (2.9%)	6 (8.6%)	

Data are presented as mean ± SD, median (interquartile range), and number (percent) where applicable.

### 3.3 Results of single factor analysis of ratio indices of CEUS quantitative parameters of thyroid nodules in the training cohort

**Figure 2:**
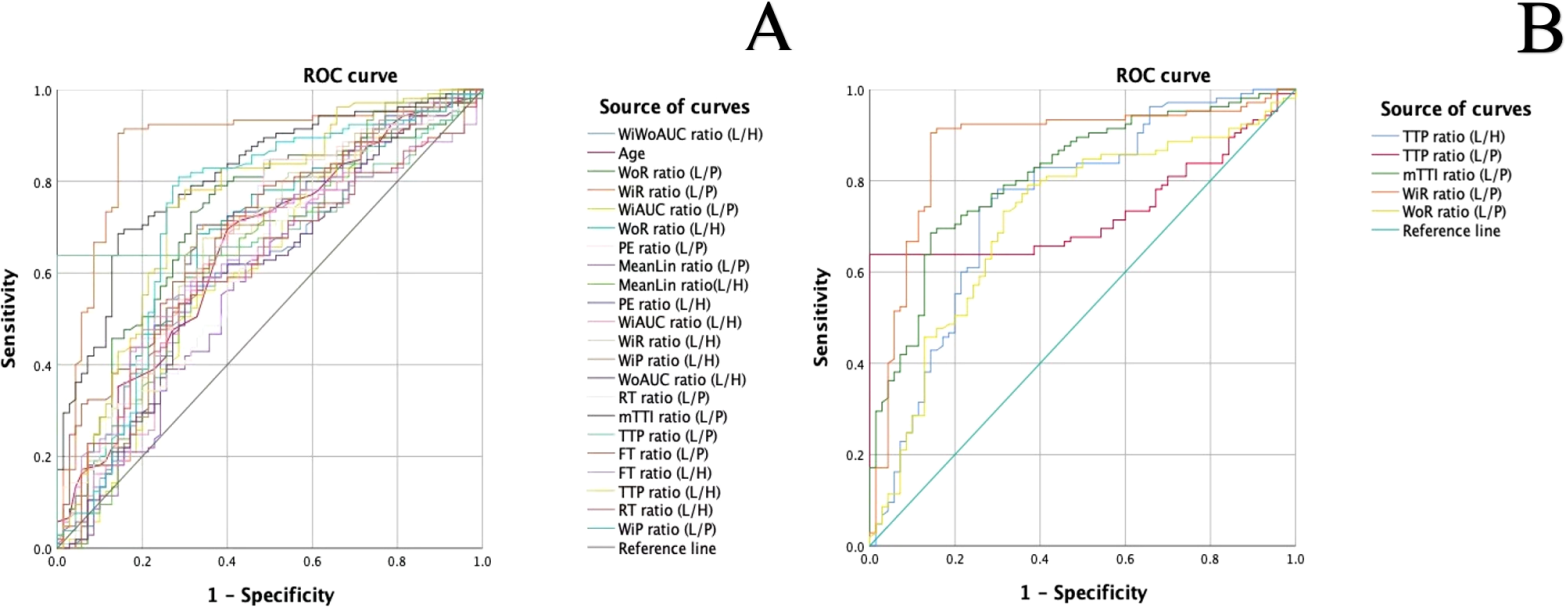
**(A)**. ROC curves of ratio indices of CEUS quantitative parameters in the diagnosis of malignant and benign thyroid nodules (L/H: the internal tissue of the lesion/the healthy tissue of the thyroid; L/P: the internal tissue of the lesion/the peripheral tissue of the thyroid nodule **(B)**. ROC curves of meaningful quantitative parameter ratios with an AUC > 0.7.

Results of single factor analysis of ratio indices of CEUS quantitative parameters of thyroid nodules were summarized in **Table 2** and the ROC curves of these parameters were presented in **Figure 2A**. Parameters with an AUC > 0.7 and a P value < 0.05 including WiR ratio (L/P) (AUC = 0.873, P<0.001), WoR ratio (L/P) (AUC = 0.714, P<0.001), TTP ratio (L/H) (AUC = 0.751, P<0.001), TTP ratio (L/P) (AUC = 0.733, P<0.001), and mTTl ratio (L/P) (AUC = 0.813, P<0.001) were further included in multifactor analysis.

**Table 2:** Ratio indices of quantitative CEUS parameters for thyroid nodules in the training cohort.

Parameter	Overall (n=175)	Malignant (n=105)	Benign (n=70)	P value
MeanLin ratio (L/H) [%]	70.02 (45.27,95.29)	56.83 (40.15,90.23)	80.94 (50.34,120.17)	0.003
PE ratio (L/H) [%]	61.56 (37.59,85.30)	46.23 (34.51,75.12)	73.32 (45.60,116.95)	<0.001
WiAUC ratio (L/H) [%]	67.38 (42.42,96.17)	59.68 (40.35,84.95)	82.09 (51.72,120.35)	0.002
RT ratio (L/H) [%]	107.63 (93.91,121.62)	111.92 (95.46,127.05)	103.27 (91.50,115.79)	0.012
mTTI ratio (L/H) [%]	129.70 (83.54,174.13)	131.25 (85.69,176.49)	117.37 (79.55,164.71)	0.454
TTP ratio (L/H) [%]	106.46 (95.53,116.58)	111.71 (104.23,122.09)	97.65 (89.14,109.38)	<0.001
WiR ratio (L/H) [%]	54.54 (33.58,88.85)	47.09 (28.63,74.51)	73.55 (46.54,133.65)	<0.001
WoAUC ratio (L/H) [%]	73.91 (39.96,112.77)	63.86 (38.91,104.46)	80.97 (49.38,135.51)	0.025
WiWoAUC ratio (L/H) [%]	73.83 (40.43,110.75)	62.68 (38.81,101.54)	80.16 (50.94,137.19)	0.016
FT ratio (L/H) [%]	109.83 (91.78,130.11)	115.34 (97.30,140.20)	100.57 (89.48,114.98)	0.003
WoR ratio (L/H) [%]	53.81 (28.08,90.39)	43.35 (25.82,67.03)	73.35 (41.76,128.97)	<0.001
MeanLin ratio (L/P) [%]	66.61 (45.38,91.24)	65.02 (45.02,84.51)	76.35 (47.97,111.50)	0.043
PE ratio (L/P) [%]	59.43 (38.50,85.96)	52.05 (36.35,75.81)	82.93 (48.79,115.76)	<0.001
WiAUC ratio (L/P) [%]	62.93 (41.88,87.09)	58.03 (40.97,80.21)	72.46 (51.55,104.41)	0.014
RT ratio (L/P) [%]	103.35 (94.07,110.36)	105.93 (98.95,118.49)	99.33 (87.80,107.43)	0.005
mTTI ratio (L/P) [%]	109.54 (91.06,148.39)	127.86 (104.57,182.98)	92.09 (72.26,106.74)	<0.001
TTP ratio (L/P) [%]	83.19 (67.51,83.19)	115.41 (68.36,128.32)	98.06 (89.37,104.20)	<0.001
WiR ratio (L/P) [%]	75.88 (43.23,75.88)	51.15 (31.44,76.70)	97.06 (85.29,133.98)	<0.001
WoAUC ratio (L/P) [%]	70.43 (45.04,94.28)	64.95 (43.97,92.21)	76.55 (49.28,114.46)	0.213
WiWoAUC ratio (L/P) [%]	67.72 (45.09,91.97)	62.64 (43.15,86.86)	73.70 (50.12,113.68)	0.103
FT ratio (L/P) [%]	107.61 (92.12,118.86)	109.33 (97.73,125.97)	95.94 (86.95,109.06)	<0.001
WoR ratio (L/P) [%]	60.97 (34.41,93.97)	50.39 (25.78,71.79)	84.15 (53.40,139.22)	<0.001

L/H means the internal tissue of the lesion/the healthy tissue of the thyroid; L/P means the internal tissue of the lesion/the peripheral tissue of the thyroid nodule.

### 3.4 Results of multifactor analysis in the training cohort

As showed in **Table 3**, there were significant difference in C-TIRADS (P = 0.044, OR = 3.747, 95% CI: 1.038–13.528), TTP ratio (L/H) (P = 0.023, OR = 1.038, 95% CI: 1.005–1.072), mTTl ratio (L/P) (P = 0.002, OR = 1.027, 95% CI: 1.010–1.043), TTP ratio (L/P) (P = 0.001, OR = 1.037, 95% CI: 1.015–1.059), and WiR ratio (L/P) (P < 0.001, OR = 0.968, 95% CI: 0.951–0.984), while enhancement degree and age had no statistically significant difference in malignant and benign thyroid nodules. The cutoff values and diagnostic performance of these meaningful parameters were detailed in **Table 4**, **Figure 2B**.

**Table 3:** Risk factors for malignant thyroid nodules in the training cohort.

Parameter		β	Univariate Analysis	
			Odds Ratio (95% CI)	P value
C-TIRADS	4a	1.321	3.747 (1.038,13.528)	0.044
	4b and 4c			
Enhancement degree	Hypo-enhancement	0.753	2.124 (0.729,6.186)	0.167
	Hyper-/Iso-enhancement			
Age	<45	0.172	1.187 (0.432,3.263)	0.739
	≥45			
TTP ratio (L/H) [%]		0.037	1.038 (1.005,1.072)	0.023
mTTI ratio (L/P) [%]		0.026	1.027 (1.010,1.043)	0.002
TTP ratio (L/P) [%]		0.036	1.037 (1.015,1.059)	0.001
WiR ratio (L/P) [%]		-0.033	0.968 (0.951,0.984)	<0.001
WoR ratio (L/P) [%]		0.017	1.018 (1.006,1.029)	0.002
Constant		-9.905	0.001	<0.001

L/H means the internal tissue of the lesion/the healthy tissue of the thyroid; L/P means the internal tissue of the lesion/the peripheral tissue of the thyroid nodule.

**Table 4:** Diagnostic performance of TTP ratio (L/H), mTTI ratio (L/P), TTP ratio (L/P), WiR ratio (L/P), WoR ratio (L/P), and the logistic model in the training cohort.

Parameter	AUC (95%CI)	Cutoff	Sensitivity [%]	Specificity [%]	Accuracy [%]	Z	P value
TTP ratio (L/H) [%]	0.751 (0.680,0.813)	105.69	74.29	74.29	74.29	4.709	<0.001
mTTI ratio (L/P) [%]	0.813 (0.749,0.877)	111.22	68.57	85.71	75.43	3.927	<0.001
TTP ratio (L/P) [%]	0.733 (0.661,0.797)	98.58	63.81	100.00	78.28	5.702	<0.001
WiR ratio (L/P) [%]	0.873 (0.815,0.919)	81.96	90.48	85.71	88.57	2.232	<0.001
WoR ratio (L/P) [%]	0.714 (0.641,0.780)	70.79	73.33	68.57	71.42	5.639	<0.001
Logistic model	0.935 (0.888,0.967)	⎯⎯	85.71	88.57	86.86	⎯⎯	<0.001

L/H means the internal tissue of the lesion/the healthy tissue of the thyroid; L/P means the internal tissue of the lesion/the peripheral tissue of the thyroid nodule.

### 3.5 Construction of the logistic regression model and assessment of its diagnostic efficacy

**Figure 3:**
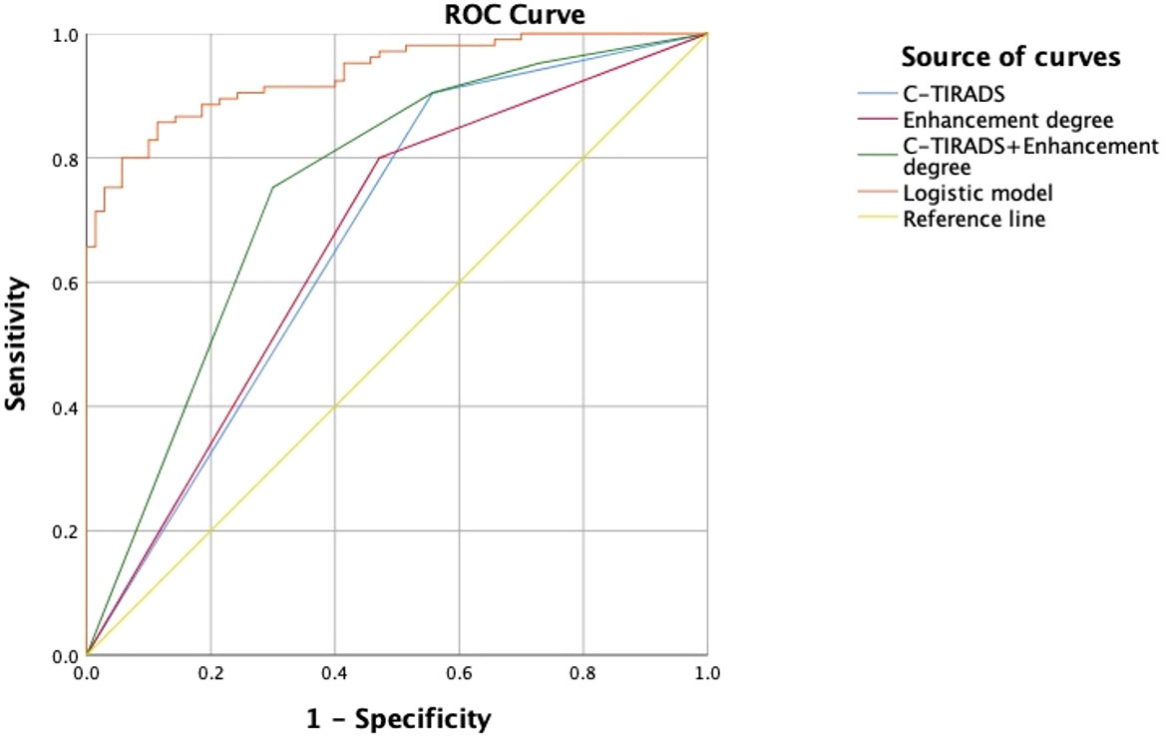
ROC curves of C-TIRADS, Enhancement degree, C-TIRADS + Enhancement degree, and the logistic model.

The logistic regression model was developed from the multifactor analysis, the logistic prediction model was developed as follows: logit (P) = -9.905 + 1.321* C-TIRADS+0.037* TTP ratio (L/H) +0.036* TTP ratio (L/P)-0.033*WiR ratio (L/P) +0.017*WoR ratio (L/P) +0.026*mTTI ratio (L/P) (0 for C-TIRADS 4a, 1 for C-TIRADS 4b and 4c). The AUC, sensitivity, specificity, and accuracy were 0.935(95% CI: 0.888–0.967),85.71%, 88.57%, and 86.86%, respectively. The comparison between the logistic model and the meaningful quantitative parameters was summarized in **Table 4**, and the comparison in the logistic model, C-TIRADS, Enhancement degree, and C-TIRADS + Enhancement degree was summarized in **Table 5**, **Figure 3**. A significant difference was observed between the logistic model and any of them alone (P < 0.001).

**Table 5:** Diagnostic performance of C-TIRADS, Enhancement degree, C-TIRADS+ Enhancement degree, and the logistic model in the training cohort.

	AUC (95%CI)	Sensitivity [%]	Specificity [%]	Accuracy [%]	Z	P value
C-TIRADS	0.671(0.596,0.740)	90.48	44.29	72.00	8.203	<0.001
Enhancement degree	0.664(0.589,0.734)	80.00	52.86	69.14	7.641	<0.001
C-TIRADS+ Enhancement degree	0.742(0.671,0.805)	74.29	70.00	72.57	5.795	<0.001
Logistic model	0.935(0.888,0.967)	85.71	88.57	86.86	⎯⎯	<0.001

### 3.6 Validation of the logistic diagnostic model

There were 60 nodules with definite pathological results, including 28 benign nodules and 32 malignant nodules, in the validation cohort (**Supplementary Tables 1**, **2**). The data of patients in the validation cohort were substituted into the logistic model to evaluate the diagnostic efficacy of the model. In the validation cohort, 32 nodules (53.3%) were pathologically malignant, and 28 nodules (46.7%) were pathologically benign, while 30 nodules were diagnosed as malignant, and 30 nodules were diagnosed as benign by the diagnostic model. The AUC, sensitivity, specificity of the diagnostic model were 0.910, 87.90%, 92.60%, respectively in the validation cohort.

**Figure 4:**
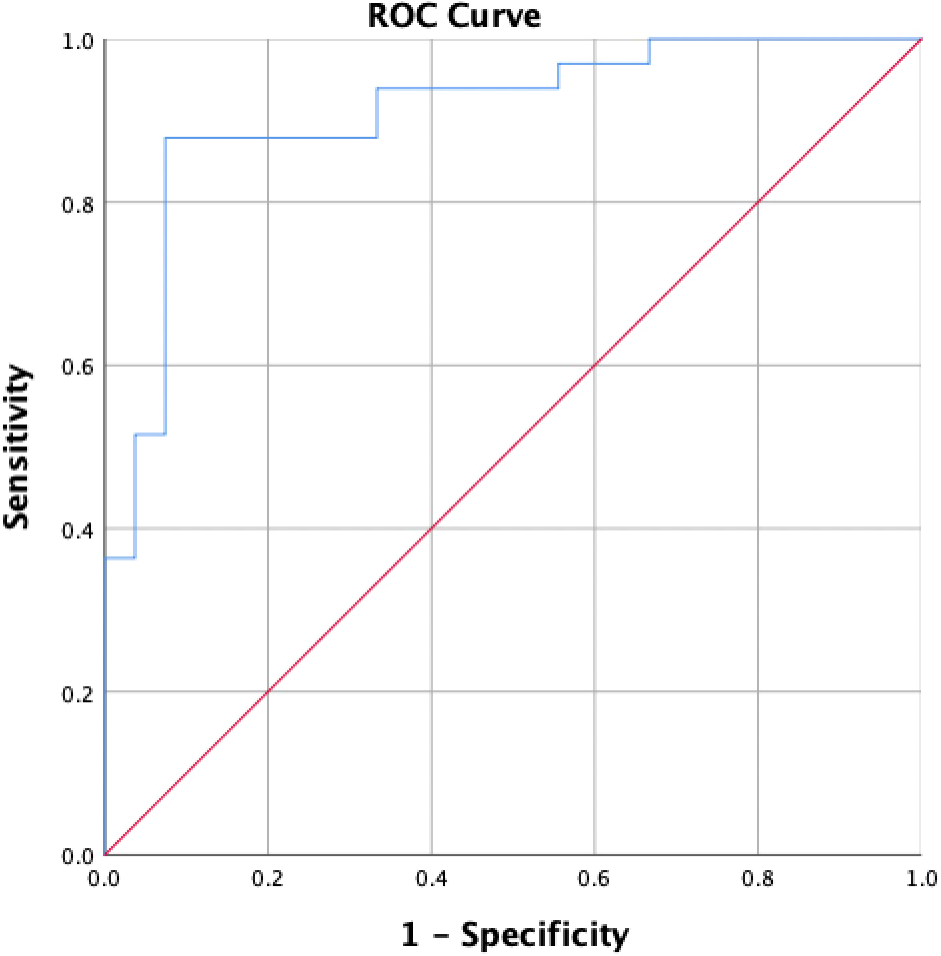
ROC curve of the logistic model in the validation cohort.

The model had significantly higher diagnostic efficacy than TTP ratio (L/H), WiR ratio (L/P), WoR ratio (L/P), C-TIRADS, Enhancement degree alone in distinguishing malignant thyroid nodules. The ROC curves of the above eight methods were compared by the Z test (**Supplementary Table 3**, **Figure 4**).

## 4 Discussion

With the rapid advancement of ultrasonic medical technology, the diagnostic efficiency for thyroid nodules has significantly improved. To standardize and summarize the malignant characteristics of thyroid nodules, various medical societies have introduced different risk stratification criteria. In China, the Chinese Thyroid Imaging Reporting and Data System (C-TIRADS) has been widely adopted (22). Hu et al. (23) conducted a systematic review and found that the prevalence of malignancies across various risk categories aligned with the classifications established by C-TIRADS. And comprehensive efficiency performance of C-TIRADS 4b were better than of C-TIRADS 4a and 4c. In addition to conventional US, contrast-enhanced ultrasound enables radiologists to assess thyroid nodules dynamically and continuously by evaluating microvascular perfusion and hemodynamic parameters. This technique has proven to be highly sensitive and effective for diagnosing malignant and benign thyroid nodules (24). In previous studies, researchers have concluded that heterogeneous enhancement, hypo-enhancement, and the absence of ring enhancement are useful for detecting malignant lesions (4, 25). However, the overlap of enhancement features between malignant and benign nodules, especially in those with a maximum diameter of ≤1.0 cm, may complicate radiologists’ assessments. In this study, the enhancement degree of malignant nodules was significantly lower than that of benign nodules (P < 0.001), while no significant differences were observed in the heterogeneity of enhancement or the centripetal enhancement pattern between malignant and benign nodules. On one hand, small tumors exhibit lower blood supply due to immature vascular bed formation and dense interstitial fibrosis, leading to hypo-enhancement on CEUS (26–28). On the other hand, small tumors tend to exhibit a more uniform distribution of blood vessels and a minimal disparity in vascular density between their central and peripheral regions. This uniformity may make radiologists to detect heterogeneity enhancement and the centripetal enhancement pattern difficultly (29, 30).

Recently, quantitative CEUS analysis has been widely utilized across various organs, offering significant value in diagnosis (16). VueBox^®^, an external offline perfusion analysis software designed to assess cine loops, facilitates a comprehensive evaluation of the dynamic wash-in and wash-out processes of microvascularity (31–34). For small thyroid nodules whose CEUS features are not significantly changed and cannot be detected by the operator, VueBox^®^ perfusion imaging technology can be used to evaluate the microvascular perfusion of the nodules to assist with diagnosis (8, 35). Moreover, previous studies have pointed out that CEUS qualitative features of peripheral tissue of thyroid tissue had potential additional value in diagnosis, but the conclusion has been still controversial. In this study, the ratio indices of quantitative parameters calculated from the time-intensity curve (TIC), including WoR ratio (L/P), mTTI ratio (L/P), TTP ratio (L/P), WiR ratio (L/P), and TTP ratio (L/H), with an AUC > 0.7, were significantly different in malignant nodules compared to benign nodules. Combining C-TIRADS with both qualitative and quantitative CEUS parameters had significantly higher accuracy compared to individual methods (P<0.001). The diagnostic model also had better diagnostic efficacy (AUC = 0.910) than the individual values in the validation group: C-TIRADS (AUC = 0.769), Enhancement degree (AUC = 0.593), TTP ratio (L/H) (AUC = 0.603), mTTI ratio (L/P) (AUC = 0.898), TTP ratio (L/P) (AUC = 0.877), WiR ratio (L/P) (AUC = 0.759), and WoR ratio (L/P) (AUC = 0.635). These results indicated that the application of CEUS quantitative analysis based on VueBox^®^ and relationship among the peripheral and internal tissue of the thyroid nodule and healthy thyroid tissue could provide useful additional information beyond subjective qualitative US and CEUS assessments in routine clinical practice.

TTP, mTTI, WoR and WiR reflect the changes in microbubble velocity and flow rate over time. Huang et al. (8] found that the WiR in malignant nodules was significantly lower than that in benign nodules (P = 0.047), with an AUC of 0.643, a sensitivity of 43.5%, and a specificity of 91.1%. Zhou et al. (15) found that the high ascending slope value were significant factor of malignancy (P < 0.05). In this study, TTP ratio (L/H), TTP ratio (L/P) and mTTI ratio (L/P) were significantly higher in malignant nodules than in benign nodules, while the WiR ratio (L/P) and WoR ratio (L/P) was significantly lower in malignant nodules compared to benign nodules. As illustrated in the ROC curves, WiR ratio (L/P) exhibited the highest diagnostic efficacy (AUC = 0.873), with a cutoff of 81.96%, a sensitivity of 90.48%, a specificity of 85.71%, and an accuracy of 88.57%. Meanwhile, mTTI ratio (L/P), TTP ratio (L/H) and TTP ratio (L/P) had an AUC of 0.813,0.751 and 0.733, respectively, with a sensitivity of 85.71%, 74.29% and 63.81%, a specificity of 85.71%, 74.29% and 100.00%, and an accuracy of 75.43%, 74.29% and 78.28%, respectively. The observed slow wash-in rate on quantitative CEUS, along with hypoechogenicity on conventional US and hypo-enhancement and centripetal enhancement on CEUS in malignant thyroid nodules, can be attributed to insufficient blood supply in thyroid carcinoma. This insufficiency may result from factors such as necrosis, potential cancer emboli that may cause vascular stenosis or blockage, as well as fibrosis and calcification (12, 15, 26). In this study, WoR ratio (L/P) was observed to be significantly lower and mTTI ratio (L/P) significantly higher in thyroid carcinoma, which was contrary to previous studies (15, 20, 26). This could be because that when the microvessels within a lesion exhibit tortuosity, and both the vein density and lumen size are diminished, alongside obstructed lymphatic reflux, particularly in the presence of numerous cancer emboli within the microvascular bed of the lesion, the contrast agent tends to accumulate within the blood vessels of the lesion. This accumulation hinders the clearance process, resulting in a lower wash-out rate and a prolonged mean transit time (36). Moreover, Outward invasion and irregular wash-out of hypovascularized margin of malignant tumor tissue may make the boundary between the tumor and the surrounding tissue unclear, and eventually lead to the observation of the object range is not so accurate (36, 37). In conclusion, the complexity of the angiogenic state of thyroid carcinoma leads to differences in the results obtained from different studies. Despite the debate over the interpretation of some indicators, in this study, the diagnostic efficiency of the logistic model surpassed that of the previous C-TIRADS classification, demonstrating that by combining qualitative and quantitative parameters, we can evaluate thyroid nodules more accurately and objectively.

There are some limitations in this study. Firstly, this was a single-center retrospective study and all of the study subjects were C-TIRADS 4 nodules, which may lead to a selection bias. Secondly, the sample size was relatively small, so verification and generalizability of our study was not clear. Finally, the current study categorized thyroid nodules solely as malignant or benign, without further delineating the various pathological types.

In the future research, we will focus on integrating CEUS with other diagnostic technologies and clinical factors to construct a multimodal diagnostic model to improve diagnostic performance. First, biochemical markers such as BRAF V600E, thyroglobulin (Tg), and specific microRNA have been proven to have potential clinical value in distinguishing malignant nodules and predicting aggressive disease course of PTC including ETE and CLNM (38–40), and it has been proved that the establishment of multimodal prediction model combined with BRAF V600E and conventional ultrasound can improve the diagnosis rate (41, 42). Second, artificial intelligence (AI) has been reported to have better diagnostic performance than human experts in thyroid differentiation and assist radiologists with diagnosis by processing ultrasonic videos and images (43–45). In the future, the research and application of machine learning and AI will further improve the accuracy of diagnoses. Moreover, we intend to undertake prospective multicenter studies with larger sample sizes and include the distinct pathological types and C-TIRADS classifications of thyroid nodules to enhance the diagnostic generalizability and practicability and reduce unnecessary FNACs and surgeries in clinical decision making.

## 5 Conclusion

Ratio indices of quantitative CEUS parameters demonstrate additional diagnostic values in distinguishing small solid C-TIRADS 4 thyroid nodules. Furthermore, the combination of C-TIRADS and CEUS parameters can improve the diagnostic efficiency, thereby providing additional value in clinical diagnosis and treatment.

## Data Availability

The original contributions presented in the study are included in the article/**Supplementary Material**. Further inquiries can be directed to the corresponding author.
