# Supplementary material for: Additional diagnostic value of ratio indices of quantitative contrast-enhanced ultrasound parameters in small solid C-TIRADS 4 thyroid nodules
Source: Front Oncol. 2025 Apr 22;15:1565400. doi: 10.3389/fonc.2025.1565400 (PMC12053176; doi:10.3389/fonc.2025.1565400)
Supplement: Supplementary file 1 [file Table1.docx]

**Supplemental Material**

**Additional diagnostic value of ratio indices of quantitative contrast-enhanced ultrasound parameters in small solid C-TIRADS 4 thyroid nodules**

Supplemental, Tables 1-3

Supplemental Table 1. Demographics and US and CEUS Qualitative parameters of thyroid nodules in the validation cohort

| Parameter |  | Overall (n=60) | Malignant (n=32) | Benign (n=28) |
| --- | --- | --- | --- | --- |
| Age,y | <45 | 40(66.7%) | 28(87.5%) | 12(42.9%) |
|  | ≥45 | 20(33.3%) | 4(12.5%) | 16(57.1%) |
| Sex | Male | 10(16.7%) | 4(12.5%) | 6(21.4%) |
|  | Female | 50(83.3%) | 28(87.5%) | 22(78.6%) |
| Size,mm |  | 7.08±0.35 | 6.48±0.41 | 7.81±0.56 |
| Laterality | Left | 28(46.7%) | 14(43.8%) | 14(50.0%) |
|  | Right | 27(45.0%) | 15(46.9%) | 12(42.9%) |
|  | Isthmus | 5(8.3%) | 3(9.3%) | 2(7.1%) |
| C-TIRADS | 4a | 20(33.3%) | 3(9.3%) | 17(60.7%) |
|  | 4b and 4c | 40(66.7%) | 29(90.7%) | 11(39.3%) |
| Enhancement degree | Hypo-enhancement | 55(91.7%) | 28(87.5%) | 27(96.4%) |
|  | Hyper-/Iso- enhancement | 5(8.3%) | 4(12.5%) | 1(3.6%) |
| Enhancement homogeneity | Homogeneous | 30(50.0%) | 13(40.6%) | 17(60.7%) |
|  | Heterogeneous | 30(50.0%) | 19(59.4%) | 11(39.3%) |
| Enhancement pattern | Centripetal | 57(94.9%) | 32(100.0%) | 25(89.3%) |
|  | Centrifugal | 3(5.1%) | 0(2.9%) | 3(11.7%) |

Data are presented as mean±SD, median (interquartile range), and number (percent) where applicable.

Supplemental Table 2. Ratio indices of Quantitative CEUS parameters for thyroid nodules in the validation cohort

| Parameter | Overall (n=60) | Malignant (n=32) | Benign (n=28) |
| --- | --- | --- | --- |
| MeanLin ratio (L/H) [%] | 59.85(46.42,80.94) | 56.15(45.54,74.12) | 76.01(47.87,99.56) |
| PE ratio (L/H) [%] | 49.58(37.72,83.73) | 41.87(36.94,61.56) | 73.86(45.02,99.47) |
| WiAUC ratio (L/H) [%] | 59.77(41.71,83.71) | 50.60(40.35,68.41) | 79.26(42.04,108.82) |
| RT ratio (L/H) [%] | 107.03(92.67,117.57) | 110.65(97.84,121.89) | 101.56(90.76,111.68) |
| mTTI ratio (L/H) [%] | 115.22(79.96,161.81) | 131.25(83.29,172.69) | 108.35(97.84,11.89) |
| TTP ratio (L/H) [%] | 105.90(94.38,116.15) | 106.04(100.41,118.42) | 102.54(92.14,114.56) |
| WiR ratio (L/H) [%] | 50.64(32.20,84.02) | 41.73(29.34,66.45) | 74.03(47.56,113.43) |
| WoAUC ratio (L/H) [%] | 63.05(40.71,97.55) | 53.29(39.49,88.52) | 78.07(44.01,142.11) |
| WiWoAUC ratio (L/H) [%] | 61.66(41.19,94.30) | 51.75(39.21,88.52) | 80.16(50.94,137.19) |
| FT ratio (L/H) [%] | 106.03(91.29,124.07) | 115.34(99.77,131.79) | 93.89(87.90,113.78) |
| WoR ratio (L/H) [%] | 52.06(25.77,89.92) | 34.73(24.88,58.83) | 76.88(45.66,127.47) |
| MeanLin ratio (L/P) [%] | 65.18(45.01,84.35) | 65.18(44.92,86.06) | 63.81(44.66,96.17) |
| PE ratio (L/P) [%] | 57.88(39.81,85.90) | 52.05(38.13,79.32) | 69.45(48.27,92.96) |
| WiAUC ratio (L/P) [%] | 61.35(45.01,84.34) | 60.48(41.78,80.69) | 62.94(49.38,90.07) |
| RT ratio (L/P) [%] | 101.99(93.80,107.16) | 102.75(96.07,107.16) | 100.03(92.33,108.41) |
| mTTI ratio (L/P) [%] | 106.02(92.45,125.37) | 119.30(106.47,150.79) | 90.98(76.26,102.81) |
| TTP ratio (L/P) [%] | 108.85(95.19,123.75) | 121.59(114.00,128.61) | 95.18(91.79,104.66) |
| WiR ratio (L/P) [%] | 74.63(46.15,96.14) | 55.80(34.81,77.33) | 88.64(75.88,108.78) |
| WoAUC ratio (L/P) [%] | 65.12(44.08,90.68) | 75.89(42.91,92.10) | 63.57(48.87,91.34) |
| WiWoAUC ratio (L/P) [%] | 63.33(44.08,88.36) | 65.00(42.54,84.16) | 63.32(49.06,91.98) |
| FT ratio (L/P) [%] | 104.93(91.45,112.92) | 108.38(98.54,125.50) | 95.61(88.96,107.73) |
| WoR ratio (L/P) [%] | 61.63(31.95,86.74) | 52.78(24.63,72.17) | 75.32(44.56,104.42) |

L/H means the internal tissue of the lesion/the healthy tissue of the thyroid; L/P means the internal tissue of the lesion/ the peripheral tissue of the thyroid nodule.

Supplemental Table 3. Comparison of Diagnostic Performance of TTP ratio (L/H), mTTI ratio (L/P), TTP ratio (L/P), WiR ratio (L/P), WoR ratio (L/P), C-TIRADS, Enhancement Degree and the logistic model in the validation cohort

| Parameter | AUC (95%CI) | Sensitivity [%] | Specificity [%] | Accuracy [%] | Z | P value |
| --- | --- | --- | --- | --- | --- | --- |
| TTP ratio (L/H) [%] | 0.603(0.456,0.749) | 81.80 | 44.45 | 63.13 | 4.325 | 0.174 |
| mTTI ratio (L/P) [%] | 0.898(0.817,0.979) | 81.80 | 81.50 | 81.65 | 0.215 | < 0.001 |
| TTP ratio (L/P) [%] | 0.877(0.779,0.974) | 78.80 | 96.30 | 87.55 | 1.870 | < 0.001 |
| WiR ratio (L/P) [%] | 0.759(0.625,0.892) | 84.80 | 74.10 | 79.45 | 2.877 | 0.001 |
| WoR ratio (L/P) [%] | 0.635(0.489,0.782) | 78.80 | 55.60 | 67.20 | 3.753 | 0.073 |
| C-TIRADS | 0.769(0.642,0.897) | 90.90 | 63.00 | 76.95 | 2.596 | < 0.001 |
| Enhancement degree | 0.593(0.445,0.740) | 100.0 | 18.50 | 59.25 | 6.664 | 0.220 |
| Logistic model | 0.910(0.834,0.986) | 87.90 | 92.60 | 90.25 | —— | < 0.001 |

L/H means the internal tissue of the lesion/the healthy tissue of the thyroid; L/P means the internal tissue of the lesion/ the peripheral tissue of the thyroid nodule.
